# Adrenomedullin binding improves catecholamine responsiveness and kidney function in resuscitated murine septic shock

**DOI:** 10.1186/2197-425X-1-2

**Published:** 2013-10-29

**Authors:** Katja Wagner, Ulrich Wachter, Josef A Vogt, Angelika Scheuerle, Oscar McCook, Sandra Weber, Michael Gröger, Bettina Stahl, Michael Georgieff, Peter Möller, Andreas Bergmann, Frauke Hein, Enrico Calzia, Peter Radermacher, Florian Wagner

**Affiliations:** Sektion Anästhesiologische Pathophysiologie und Verfahrensentwicklung, Klinik für Anästhesiologie, Ulm, 89081 Germany; Institut für Pathologie, Universitätsklinikum, Albert-Einstein-Allee 23, Ulm, 89081 Germany; Adrenomed AG, Hennigsdorf, 16761 Germany

**Keywords:** Cecal ligation and puncture, Creatinine clearance, Neutrophil gelatinase-associated lipocalin, Inducible nitric oxide synthase, Endothelial nitric oxide synthase, Peroxynitrite, Apoptosis

## Abstract

**Purpose:**

Adrenomedullin (ADM) has been referred to as a *double-edged sword* during septic shock: On one hand, ADM supplementation improved organ perfusion and function, attenuated systemic inflammation, and ultimately reduced tissue apoptosis and mortality. On the other hand, ADM overproduction can cause circulatory collapse and organ failure due to impaired vasoconstrictor response and reduced myocardial contractility. Since most of these data originate from un-resuscitated shock models, we tested the hypothesis whether the newly developed anti-ADM antibody HAM1101 may improve catecholamine responsiveness and thus attenuate organ dysfunction during resuscitated murine, cecal ligation and puncture (CLP)-induced septic shock.

**Methods:**

Immediately after CLP, mice randomly received vehicle (phosphate-buffered saline, *n* = 11) or HAM1101 (*n* = 9; 2 μg·g^−1^). Fifteen hours after CLP, animals were anesthetized, mechanically ventilated, instrumented, and resuscitated with hydroxyethylstarch and continuous i.v. norepinephrine to achieve normotensive hemodynamics (mean arterial pressure > 50 to 60 mmHg).

**Results:**

HAM1101 pretreatment reduced the norepinephrine infusion rates required to achieve hemodynamic targets, increased urine flow, improved creatinine clearance, and lowered neutrophil gelatinase-associated lipocalin blood levels, which coincided with reduced expression of the inducible nitric oxide synthase and formation of peroxynitrite (nitrotyrosine immunostaining) in the kidney and aorta, ultimately resulting in attenuated systemic inflammation and tissue apoptosis.

**Conclusions:**

During resuscitated murine septic shock, early ADM binding with HAM1101 improved catecholamine responsiveness, blunted the shock-related impairment of energy metabolism, reduced nitrosative stress, and attenuated systemic inflammatory response, which was ultimately associated with reduced kidney dysfunction and organ injury.

**Electronic supplementary material:**

The online version of this article (doi:10.1186/2197-425X-1-2) contains supplementary material, which is available to authorized users.

## Introduction

Adrenomedullin (ADM) has been referred to as a *double-edged sword* in sepsis [1]: during cecal ligation and puncture (CLP)- [2–4], endotoxin- [5], or *Staphylococcus aureus* α-toxin-induced sepsis [6], ADM administration restored organ perfusion during the hypodynamic shock phase. This effect coincided with attenuated systemic inflammation [7], decreased activation of the inducible nitric oxide synthase (iNOS) and peroxynitrite formation [8], and reduced organ dysfunction [9], apoptosis [10], and mortality [6]. ADM release is therefore referred to as a protective adaption to systemic inflammation [11] and circulatory shock [12]. Nevertheless, its overproduction may cause circulatory collapse and organ failure [13] due to peripheral vasodilation [14]: arterial hypotension and reduced systemic vascular resistance correlated with increased ADM concentrations in volunteers injected with endotoxin [15] and patients with sepsis [16]. Moreover, ADM levels were directly related to morbidity and mortality [17–21], and persistently high concentrations allowed predicting infectious complications after septic shock [22]. The ADM-related hypotension results from excess NO release with impaired vasoconstrictor response [23, 24] and myocardial contractility [25]. In un-resuscitated shock, ADM antagonists reversed these effects and ultimately improved survival [24, 25]. Hollenberg et al., however, showed that survival of wild-type and iNOS^−/−^ mice after CLP-induced sepsis only differed when adequate resuscitation was provided [26]. Therefore, we tested the hypothesis whether a newly developed ADM antibody may improve catecholamine responsiveness and attenuate kidney dysfunction in resuscitated, CLP-induced septic shock [27].

## Materials and methods

### Anesthesia, surgical instrumentation, and experimental protocol

The study protocol was approved by the University Animal Care Committee and the federal authorities for animal research of the Regierungspräsidium Tübingen, Baden-Württemberg. A total of 23 male C57BL/6J mice (body weight 23 to 29 g, age 10 to 16 weeks) were used for the experiments, which were performed in adherence to the National Institutes of Health Guidelines on the Use of Laboratory Animals. Animals which did not undergo mechanical ventilation and surgical instrumentation served as controls for the assessment of blood ADM levels (*n* = 4) and tissue immune blotting and gel shifts (*n* = 6). Immediately after CLP, mice randomly received vehicle (phosphate-buffered saline (PBS), *n* = 11) or the anti-ADM antibody HAM1101 (*n =* 12; 2 μg·g^−1^ to achieve antibody concentrations >4 μg·mL^−1^, i.e., to guarantee plasma antibody levels two orders of magnitude in excess of ADM concentrations). HAM1101 is an antibody directed to the N-terminal part of ADM and was chosen because, in pilot experiments, it had significantly improved 14-day survival after CLP in mice (*n* = 10) with otherwise 100% mortality within two (vehicle PBS) and three (control antibody) days. Three animals were excluded: at necropsy and organ removal, one animal presented with pronounced intra-abdominal and retroperitoneal bleeding, and in the two other animals, by accident, the antibody preparation was not adequately stored within the specifications. Therefore, statistical analysis in the HAM1101 group refers to *n* = 9. The anesthesia, CLP procedure, and the surgical instrumentation have been described in detail previously [27]. Mice were anesthetized with sevoflurane and s.c. buprenorphine together with acetated Ringer's solution containing glucose. A midline laparotomy cecum ligation and a single puncture (18-gauge needle) were performed. After squeezing to expel a stool, the cecum was returned into the abdominal cavity. Postoperatively, water and food were provided *ad libitum*. After 8 h, mice received a second s.c. injection as described above together with buprenorphine, ceftriaxon, and clindamycine. Fifteen hours post-CLP, mice were anesthetized with sevoflurane, i.p. ketamine, midazolam, and fentanyl. After placement of the animal on the procedure bench equipped with heating pads and lamps, a rectal temperature probe was inserted. The anterior neck was incised to expose the trachea, the right internal jugular vein, and the right carotid artery. The trachea was intubated, and the lungs were mechanically ventilated with a pressure-controlled, lung-protective ventilation strategy using a small animal ventilator (FlexiVent^TM^, Scireq®, Montreal, Canada) [28]. After a lung recruitment maneuver, respirator settings were as follows: FiO_2_ = 0.5, tidal volume = 6 to 8 μL·g^−1^, respiratory rate = 160 breaths·min^−1^, inspiratory/expiratory time ratio = 1:2, and PEEP = 5 cm H_2_O. Catheters were inserted into the jugular vein, the carotid artery, and the bladder. Anesthesia was maintained with continuous i.v. ketamine, fentanyl, and midazolam, titrated to reach deep sedation and analgesia as documented by complete tolerance against noxious stimuli. Normoglycemia was maintained by i.v. glucose (stable, non-radioactive labeled 1,2,3,4,5,6-^13^C_6_-glucose and unlabeled glucose, 0.5 mg·g^−1^·h^−1^ each). Normotensive hemodynamics (i.e., mean arterial pressure >50 to 60 mmHg) were achieved by i.v. hydroxyethylstarch (maximum infusion rate 20 μL·g^−1^·h^−1^) and, if needed, together with norepinephrine. After 5 h, animals were killed through blood withdrawal via the V. cava inferior.

### Nitrate, cytokine, and chemokine concentrations

As a surrogate for NO production, plasma nitrite + nitrate (NO_2_^−^+NO_3_^−^) levels were measured using chemiluminescence after reduction to NO with vanadium chloride (NOA 280 NO Analyzer, Sievers Medical Instruments, Boulder, CO, USA) [29]. Plasma tumor necrosis factor (TNF)-α, interleukin (IL)-6, monocyte chemoattractant protein (MCP)-1, and keratinocyte-derived chemokine (KC) concentrations were determined using a mouse multiplex cytokine kit (Bio-Plex Pro Cytokine Assay, Bio-Rad, Hercules, CA, USA). The assay was performed by using the Bio-Plex suspension array system according to the manufacturer's instructions [27, 28]. Levels below the detection limit of the assays were set to zero for statistical purposes.

### Adrenomedullin assay and adrenomedullin antibody (HAM1101) preparation

The detailed description is presented as an Additional file 1. Briefly, plasma ADM (both free and antibody-bound ADM in the HAM1101 group) concentrations were measured with a sandwich coated tube luminescence immunoassay, based on acridinium NHS-ester labeling and anti-mouse ADM antibodies (MAM). Dilutions of mouse adrenomedullin (mADM) and subsequently the labeled tracer MAM1301 were added to MAM1201-coated tubes. After washing of unbound tracer, chemiluminescence was measured (AutoLumat LB 953 Berthold Technologies, Bad Wildbad, Germany). For antibody preparation, murine and human ADM peptides were synthesized (JPT Technologies, Berlin, Germany) with an additional N-terminal cysteine (if no cysteine is present within the selected adrenomedullin sequence) residue for conjugation of the peptides to bovine serum albumin using SulfoLink-coupling gel (Perbio Science, Bonn, Germany). HAM1101 blood concentrations were quantified via competitive immunoassay employing chemoluminescence-labeled HAM1101 as tracer and ADM in the solid phase.

### Creatinine, urea, and neutrophil gelatinase-associated lipocalin

Blood neutrophil gelatinase-associated lipocalin (NGAL) concentrations were measured using a commercial ELISA (mouse NGAL, RUO 042, BioPorto Diagnostics A/S, Gentofte, Denmark). Urea and creatinine concentrations were quantified with a capillary column (Optima-5MS, Macherey-Nagel, Düren, Germany) gas chromatography/mass spectrometry system (Agilent 5890/5970, Böblingen, Germany) using ^2^H_3_-creatinine (CDN isotopes, Pointe-Claire, QU, Canada) and methylurea (Fluka Chemikalien, Buchs, Switzerland) as internal standards. After deproteinization with acetonitrile and evaporation to dryness, the supernatant was reconstituted in formic acid and extracted over a weak anion exchange column (WCX, Phenomenex, Aschaffenburg, Germany). Acetonitrile plus *N*-(*tert*-butyldimethylsilyl)-*N*-methyltrifluoroacetamide and *N*,*O*-bis(trimethylsilyl)trifluoroacetamide allowed formation of the urea *tert*-butyl-dimethylsilyl- and the creatinine trimethylsilyl-derivatives, respectively. Ions m/z 231 and 245, and m/z 329 and 332 were monitored for urea and creatinine analytes and internal standards. Creatinine clearance was derived from urine output and plasma and urine creatinine concentrations.

### Glucose metabolism

Parameters of glucose metabolism were determined using gas chromatography/mass spectrometry [30]. After conversion of glucose to a penta-trifluoroacetyl derivative, endogenous glucose production rate was calculated from the blood ^13^C_6_-glucose isotope enrichment. Whole-body glucose oxidation was derived from the total CO_2_ production and the ^13^CO_2_/^12^CO_2_ isotope ratio in the mixed expiratory gas.

### Cell extracts, electrophoretic mobility shift assay, immunoblots, and comet assay

Immediately postmortem, the right kidney was removed, homogenized, and lysed in lysing buffer. Cells were resuspended and lysed on ice. To assess the expression of heme oxygenase-1 (HO-1), activated caspase-3, and BcL-xL, equal total protein aliquots (20 to 60 μg) were separated by SDS-PAGE and transferred by Western blotting. After blocking, the membranes were incubated with primary antibodies (anti HO-1, Stressgen; anti-Bcl-xL, anti-cleaved caspase-3, Cell Signaling, Danvers, MA, USA). The primary antibodies were detected using horseradish peroxidase-conjugated secondary antibodies (Cell Signaling or Santa Cruz, Heidelberg, Germany). The membranes were subjected to chemiluminescence using the SuperSignal West Femto Maximum Sensitivity Substrate (Thermo Scientific, Ulm, Germany). Exposed films were scanned, and intensity of immunoreactivity was measured using NIH ImageJ software (http://rsb.info.nih.gov/nih-image). Activation of the nuclear transcription factor κB (NF-κB) was determined using an electrophoretic mobility shift assay (EMSA) [27, 28]: cell extracts were incubated with poly-doxy-inosinic-deoxy-cytidylic acid (poly-dI-dC) and ^32^P-labeled double stranded oligonucleotide containing the NF-κB (HIVκB-site) (5′-GGATCCTCAACAGAGGGGACTTTCCGAGGCCA-3′). Complexes were separated in polyacrylamide gels and exposed to X-ray films. A phosphorimager and image analysis software allowed quantifying the radioactively labeled NF-κB. Relating the intensity of each band to that of control animals loaded simultaneously allowed for comparison between individual gels. Protein expression and NF-κB activation are presented as *x*-fold increase over control values.

Single cell gel electrophoresis allowed assessing the oxidative deoxyribonucleic acid (DNA) damage (‘tail moment’ in the alkaline version of the comet assay) [30]. Immediate postmortem biopsies were placed in buffer containing Na-ethylenediaminetetraacetic acid and minced to obtain a cell suspension. Two agarose gel slides were prepared from each biopsy. The mean tail moment of 100 nuclei analyzed per slide was used for each animal.

### Histology

Immediately postmortem, the left kidney was removed, sectioned by a sagittal cut, and fixed in paraformaldehyde for histopathological examination by an experienced pathologist (A.S.) blinded for the sample grouping. Paraffin sections were stained with hematoxylin and eosin and periodic acid-Schiff staining; photomicrographs were taken of three sampling areas from each section. Glomerular histopathological alterations were analyzed for *glomerular tubularization*, i.e., the herniation of proximal tubular epithelial cells into Bowman's capsule [31]. Data are expressed as the number of glomeruli showing glomerular tubularization in percentage of all glomeruli analyzed (outer cortical region 30, medullar region 20 glomeruli) of the three random sections. Tubular histopathological damage was assessed as intraluminal protein cylinders and necrosis.

### Immunohistochemistry for nitrotyrosine and the inducible nitric oxide synthase

The descending thoracic aorta was excised down to the diaphragm, fixed in paraformaldehyde, and embedded in paraffin. Sections of the aorta and the left kidney were dewaxed in xylene, rehydrated with ethanol, incubated in citrate buffer, brought to boiling for heat-induced antigen retrieval, and blocked with goat sera before incubation with rabbit anti-nitrotyrosine 1:250 (Millipore #-AB5411, Schwalbach, Germany), anti-eNOS (eNOS BD# 610297, Beckton Dickinson, Heidelberg, Germany), and anti-iNOS (Santa Cruz #sc-651). Primary antibody detection was performed with anti-rabbit Dako Real detection system alkaline phosphatase red (Dako #K5005, Copenhagen, Denmark). Slides were visualized using a Zeiss Axio Imager A1 microscope. Five artery and three kidney sections were quantified for intensity and area of the immune-reactive regions using the AxioVision (rel. 4.8) software (Zeiss, Jena, Germany). Results are presented as arbitrary units of densitometric sum red.

### Statistical analysis

All data are presented as median (range). After exclusion of normal distribution using the Kolmogorov-Smirnov test, differences between groups were analyzed with a one-way Kruskal-Wallis analysis of variance on ranks followed by a post hoc Dunn test. Because of the multiple statistical testing resulting from the numerous variables measured, all results have to be interpreted in an exploratory rather than confirmatory manner.

## Results

**Figure 1:**
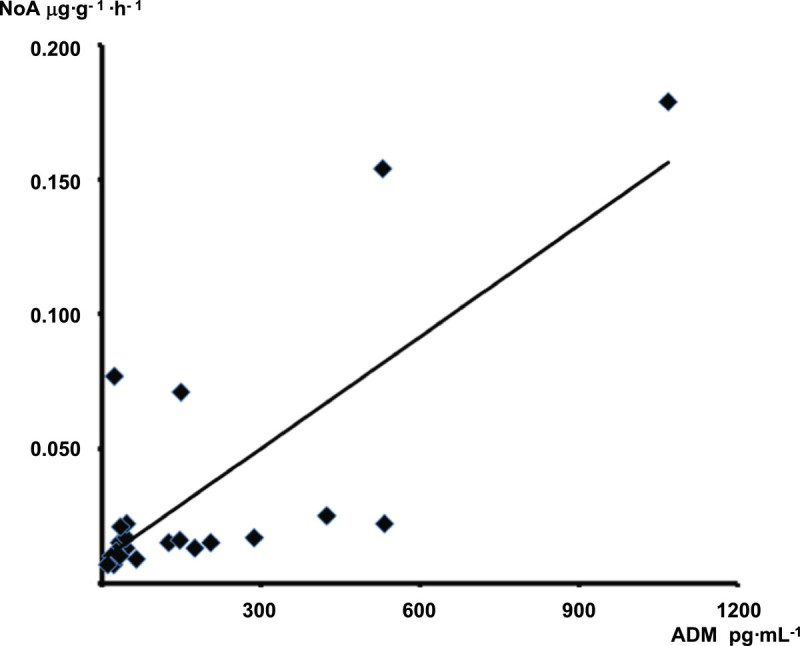
**Mean noradrenaline (NoA, μg·g**^**−1**^ **·h**^**−1**^ **) infusion rates.** The infusion rates were plotted as a function of the blood adrenomedullin (ADM, pg·mL^**−** 1^) concentrations in all 23 animals taken together, i.e., also taking into account the three animals excluded from the detailed statistical analysis because of abdominal bleeding and inadequate antibody activity. The Pearson coefficient of correlation was *r*^2^ = 0.574 (*p* < 0.001).

Plasma ADM concentrations in mice without mechanical ventilation and surgical instrumentation were 7 (4, 11) pg·mL^−1^. HAM1101 tended to attenuate the CLP-induced increase in ADM levels, however, without reaching statistical significance (*p* = 0.094; Table 1). The total, i.e., crystalloid plus colloid, fluid infusion rate was identical (vehicle 48 (47, 50), HAM1101 47 (46. 49) μL·g^−1^·h^−1^, *p* = 0.185) in the two groups. All animals needed continuous i.v. noradrenaline to achieve hemodynamic targets, and HAM1101 was associated with significantly lower mean infusion rates during the 5-h observation period (0.010 (0.09, 0.012) vs. 0.019 (0.016, 0.24) μg·g^−1^·h^−1^, *p* < 0.001). In all 23 animals taken together, i.e., also taking into account the three animals excluded from the detailed statistical analysis, there was a significant linear relationship between the ADM blood levels and the noradrenaline infusion rates (Pearson coefficient of correlation *r*^2^ = 0.574, *p* < 0.001) (Figure 1).

**Table 1:** **Plasma concentrations of mADM and HAM1101 in vehicle- (** ***n*** **= 11) and HAM1101-treated (** ***n*** **= 9) animals**

	Vehicle	HAM1101	***p*** value
mADM (pg·mL^−1^)	96 (37, 190)	33 (21, 64)	0.094
HAM1101 (μg·mL^−1^)	Ø	5.0 (4.2, 6.5)	<0.001

Blood samples were taken at the end of the experiment. All data are median (quartiles).

Hemodynamics, gas exchange, acid-base status, glycemia, endogenous glucose, or total CO_2_ production did not differ between the two groups (Table 2). However, HAM1101 lowered lactatemia and increased direct, aerobic glucose oxidation until the end of the experiment. HAM1101 was associated with three- and twofold higher diuresis and creatinine clearance, (Table 3), respectively, ultimately resulting in lower creatinine, urea, and NGAL concentrations. HAM1101 caused a two- to tenfold reduction of the blood levels of IL-10 and the pro-inflammatory cytokines and chemokines, whereas NO_2_^−^ + NO_3_^−^ concentrations did not differ (Table 4).

**Table 2:** **Lung mechanics, hemodynamics, gas exchange, acid-base status, and metabolic parameters**

		Vehicle	HAM1101	***p*** value
Hemoglobin content (mg·mL^−1^)	Start	90 (87, 96)	98 (95, 105)	0.074
	End	70 (66, 72)	69 (66, 74)	0.806
Heart rate (beats·min^−1^)	Start	494 (485, 515)	430 (406, 480)	0.248
	End	426 (406, 445)	391 (349,430)	0.290
Mean arterial pressure (mmHg)	Start	69 (65, 71)	69 (66, 76)	0.382
	End	64 (59, 69)	67 (66, 72)	0.441
Thoracopulmonary compliance (μL·cm H_2_O^−1^)	Start	0.10 (0.09, 0.10)	0.10 (0.09, 0.11)	0.382
	End	0.09 (0.08, 0.10)	0.10 (0.08, 0.11)	0.427
Arterial PO_2_ (mmHg)	Start	314 (293, 330)	340 (321, 350)	0.254
	End	309 (287, 326)	301 (287, 319)	0.838
Arterial PCO_2_ (mmHg)	Start	31 (27, 33)	31 (29, 36)	0.879
	End	34 (33, 36)	31 (28, 33)	0.072
Arterial pH	Start	7.33 (7.30, 7.35)	7.36 (7.32, 7.38)	0.569
	End	7.38 (7.31, 7.43)	7.43 (7.39, 7.46)	0.253
Arterial base excess (mmol·mL^−1^)	Start	−9.0 (−10.3, −7.7)	−6.8 (−8.3, −6.2)	0.403
	End	−5.7 (−7.1, −1.7)	−3.9 (−4.2, −3.3)	0.838
Arterial lactate (mmol·mL^−1^)	Start	n.d.	n.d.	n.d.
	End	2.0 (1.7, 2.2)	1.6 (1.5, 1.8)	0.025
Arterial glucose (mg·dL^−1^)	Start	113 (110, 117)	115 (106, 123)	0.540
	End	110 (96, 117)	111 (109, 115)	0.653
CO_2_ production rate (μL·g^−1^·min^−1^)	Start	26 (24, 28)	24 (23, 25)	0.082
	End	23 (21, 26)	23 (22, 25)	0.596
Glucose production rate (mg·g^−1^·h^−1^)	Start	0.37 (0.35, 0.41)	0.34 (0.31, 0.36)	0.178
	End	0.38 (0.35, 0.39)	0.33 (0.31, 0.39)	0.153
Glucose oxidation rate (% isotope infusion)	Start	58 (55, 62)	55 (50, 59)	0.224
	End	57 (54, 60)	62 (58, 67)	0.047

The data are for the vehicle-treated (*n* = 11) and HAM1101-treated (*n* = 9) animals at the start and the end of the 5-h observation period; n.d., not determined. All data are median (quartiles).

**Table 3:** **Parameters of kidney function in the vehicle- (** ***n*** **= 11) and HAM1101-treated (** ***n*** **= 9) animals**

	Vehicle	HAM1101	***p*** value
Urine output (μL·g^−1^·h^−1^)	4.4 (3.5, 16.5)	15.2 (13.9, 22.5)	0.033
Creatinine clearance (μL·min^−1^)	197 (110, 301)	400 (316, 509)	0.006
Creatinine (μg·mL^−1^)	1.83 (1.52, 3.04)	1.28 (1.20, 1.52)	0.010
Urea (μg·mL^−1^)	378 (268, 513)	175 (101, 184)	0.004
NGAL (μg·mL^−1^)	16 (15, 20)	11 (10, 13)	0.008

Blood concentrations were measured in samples taken at the end of the experiment. NGAL, neutrophil gelatinase-associated lipocalin. All data are median (quartiles).

**Table 4:** **Blood nitrite + nitrate (NO**_**2**_^**−**^ **+ NO**_**3**_^**−**^ **, in nmol·mL** ^**−1**^ **), cytokine, and chemokine concentrations**

	Vehicle	HAM1101	***p*** value
NO_2_^**−**^ + NO_3_^**−**^	111 (59, 121)	92 (47, 93)	0.170
TNF-α	32 (13, 62)	1 (0, 8)	0.010
IL-6	363 (120, 681)	43 (22, 55)	0.002
IL-10	33 (10, 81)	3 (2, 6)	0.004
KC	2923 (1873, 7138)	650 (516, 1577)	0.016
MCP-1	1130 (779, 1738)	509 (335, 614)	0.005

The data are for vehicle-treated (*n* = 11) and HAM1101-treated (*n* = 9) animals. Blood samples were taken at the end of the experiment. TNF-α, tumor necrosis factor-α; IL-6, interleukin-6; IL-10, interleukin-10; KC, keratinocyte-derived chemokine; MCP-1, monocyte chemoattractant protein-1. All parameters are in pg·mL^−1^. All data are median (quartiles).

**Figure 2:**
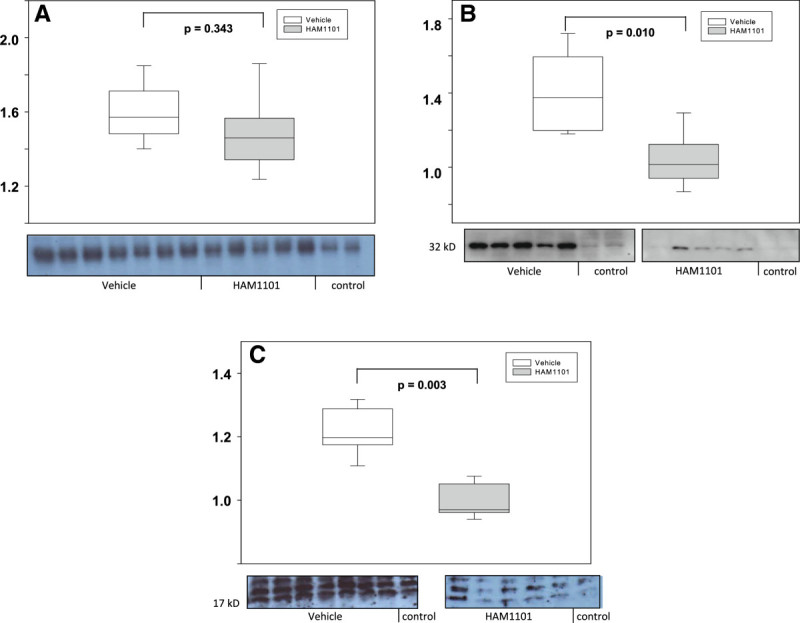
**Representative gel shifts and immune blots.** Also included is the quantitative analysis of the activation of the nuclear transcription factor κB (NF-κB) **(A)** and the tissue expression of heme oxygenase-1 (HO-1) **(B)** and cleaved caspase-3 **(C)** from immediate postmortem kidney specimen in the vehicle-treated (open box plots, *n* = 11) and HAM1101-treated (grey box plots, *n* = 9) mice. Data reported are median, quartiles, and range and refer to fold increase of values from animals which had only undergone surgical instrumentation (‘control’).

**Figure 3:**
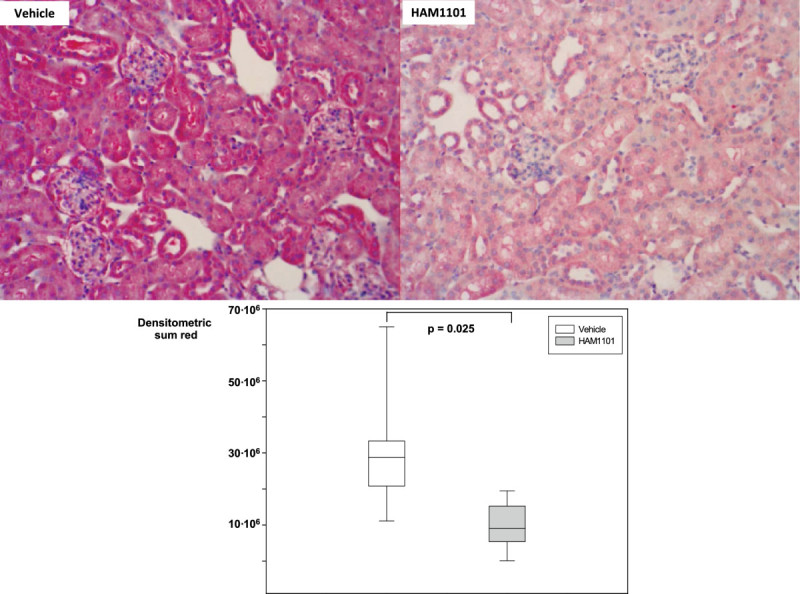
**Renal iNOS immunostaining.** Examples (magnification ×20) of the renal iNOS immunostaining from the immediate postmortem kidney specimen in the vehicle (upper left panel) and HAM1101 (upper right panel) as well as results of the quantitative image analysis (vehicle group: open box plots, *n* = 11; HAM1101 group: grey box plots, *n* = 9). Data reported are median, quartiles, and range.

**Figure 4:**
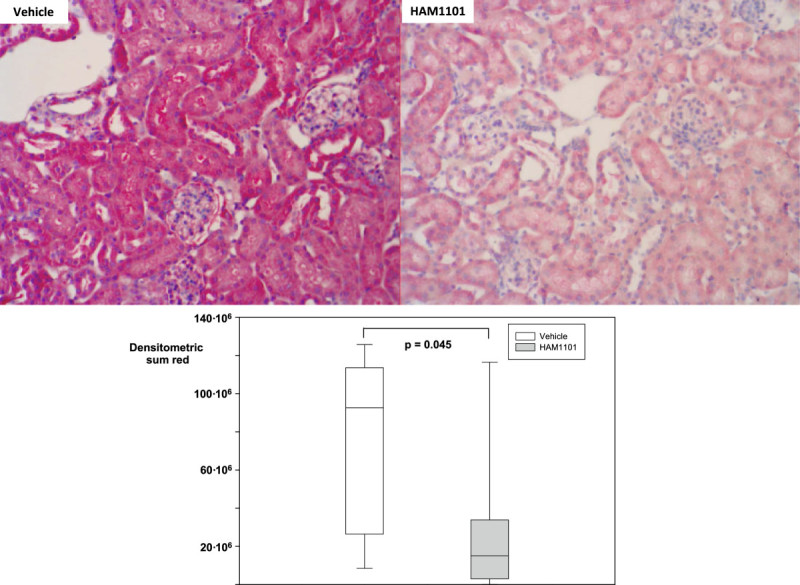
**Renal nitrotyrosine immunostaining.** Examples (magnification ×20) of the renal nitrotyrosine immunostaining from the immediate postmortem kidney specimen in the vehicle (upper left panel) and HAM1101 (upper right panel) as well as results of the quantitative image analysis (vehicle group: open box plots, *n* = 11; HAM1101 group: grey box plots, *n* = 9). Data reported are median, quartiles, and range.

**Figure 5:**
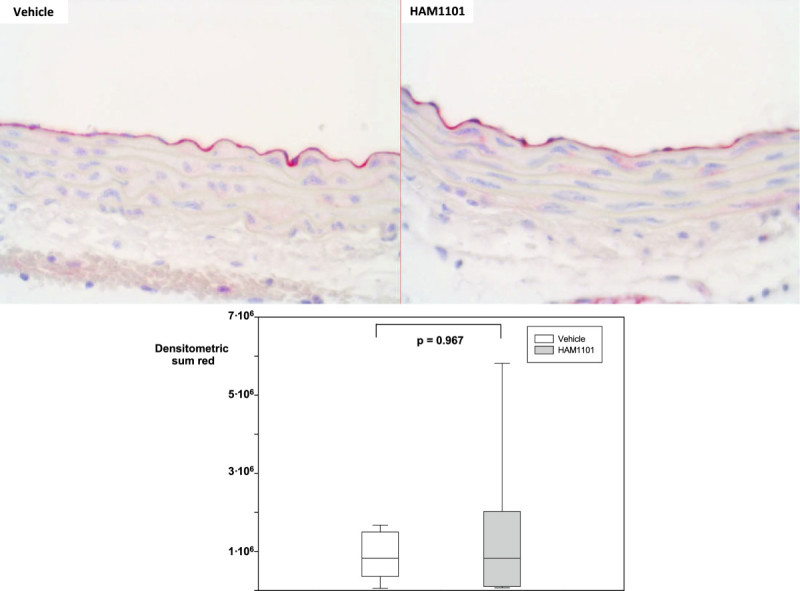
**Thoracic aortic eNOS immunostaining.** Examples (magnification ×40) of the thoracic aortic eNOS immunostaining from the immediate postmortem kidney specimen in the vehicle (upper left panel) and HAM1101 (upper right panel) as well as results of the quantitative image analysis (intima layer; vehicle group: open box plots, *n* = 11; HAM1101 group: grey box plots, *n* = 9). Data reported are median, quartiles, and range.

**Figure 6:**
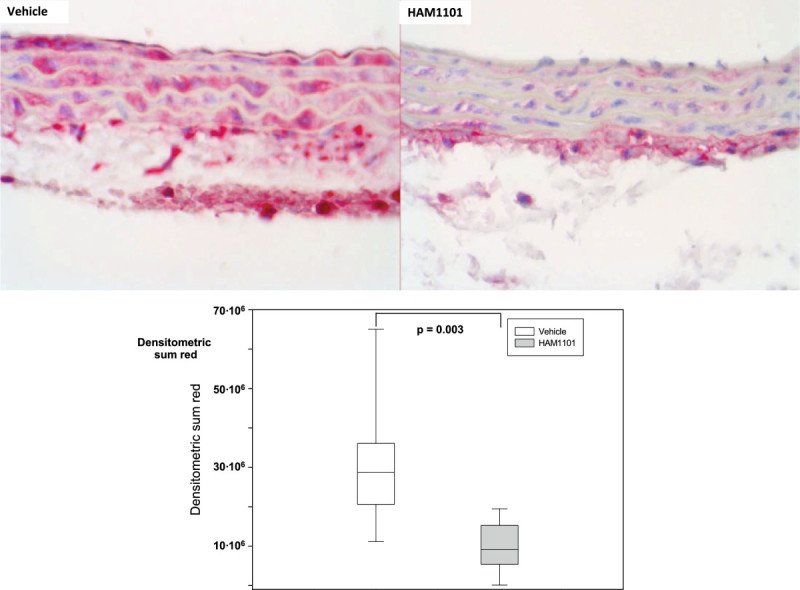
**Thoracic aortic iNOS immunostaining.** Examples (magnification ×40) of the thoracic aortic iNOS immunostaining from the immediate postmortem kidney specimen in the vehicle (upper left panel) and HAM1101 (upper right panel) as well as results of the quantitative image analysis (intima plus media layer; vehicle group: open box plots, *n* = 11; HAM1101 group: grey box plots, *n* = 9). Data reported are median, quartiles, and range.

**Figure 7:**
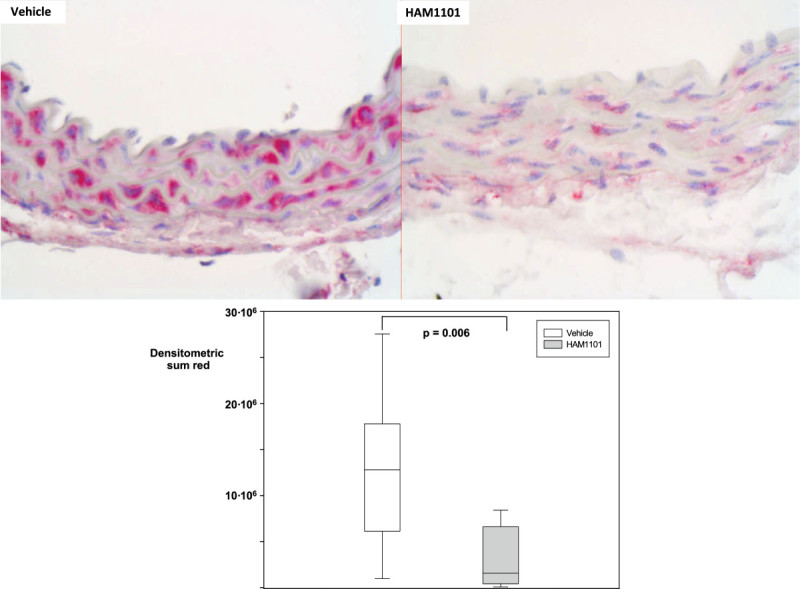
**Thoracic aortic nitrotyrosine immunostaining.** Examples (magnification ×40) of the thoracic aortic nitrotyrosine immunostaining from the immediate postmortem kidney specimen in the vehicle (upper left panel) and HAM1101 (upper right panel) as well as results of the quantitative image analysis (intima plus media layer; vehicle group: open box plots, *n* = 11; HAM1101 group: grey box plots, *n* = 9). Data reported are median, quartiles, and range.

Figure 2 shows the results of immune blotting and gel shifts: while NF-κB activation (Figure 2A) was comparable, HAM1101 attenuated the expression of HO-1 (Figure 2B) and activated caspase 3 (Figure 2C). In contrast, Bcl-xL expression and the tail moment in the comet assay did not differ (*p* = 0.149 and 0.527, respectively; data not shown). Figures 3 and 4 present typical kidney iNOS (Figure 3, upper panel) and nitrotyrosine (Figure 4, upper panel) immunostainings. Quantitative analysis (lower panel) showed that HAM1101 attenuated iNOS activation and nitrotyrosine formation. Figures 5, 6, and 7 present typical aortic eNOS (Figure 5, upper panel), iNOS (Figure 6, upper panel), and nitrotyrosine (Figure 7, upper panel) immunostainings. Image analysis demonstrates that HAM1101 attenuated iNOS activation and nitrotyrosine formation (Figures 6 and 7, lower panel), whereas eNOS expression was comparable (Figure 5, lower panel).

There was only minor kidney histological damage: glomerular tubularization was moderate without intergroup difference (vehicle: 13 (12, 14), HAM1101: 13 (9, 15) % of glomerula analyzed, *p* = 0.724), and neither tubular protein cylinders nor necrosis were found.

## Discussion

The aim of this study was to test the hypothesis whether the ADM antibody HAM1101 may improve catecholamine responsiveness and attenuate kidney dysfunction during resuscitated murine CLP-induced septic shock. The major findings were that HAM1101 (1) reduced the catecholamine infusion rates needed to maintain target hemodynamics and (2) improved renal function, which was associated with (3) attenuated systemic inflammation, iNOS activation, and peroxynitrite formation, (4) ultimately resulting in reduced apoptosis.

In the HAM1101-treated mice, the lower noradrenaline requirements coincided with reduced aortic immunostaining for iNOS and nitrotyrosine, whereas eNOS expression was unchanged. This finding fits well with previous studies on the interaction of iNOS and ADM: in vascular smooth muscle cells, ADM aggravated the cytokine-induced iNOS activation and the excess NO release [23]. *In vivo* ADM increased myocardial and coronary endothelial nitrotyrosine formation, which was prevented by the NOS inhibitor N^G^-nitro-l-arginine [32]. ADM antagonism reversed the lipopolysaccharide (LPS)-induced hypotension in rats [24], ultimately resulting in prolonged survival [25]. Furthermore, during rodent and human endotoxemia, hemodynamic stabilization with activated protein C was associated with lower blood NO and ADM levels [15]. Finally, we showed in CLP-induced septic shock that genetic deletion and selective pharmacological inhibition of iNOS improved catecholamine responsiveness: maintenance of target hemodynamics was achieved using lower noradrenaline infusion rates [29]. Nevertheless, our current findings may be regarded as in contrast to those of Talero et al. [8], who reported that ADM dose-dependently reduced iNOS expression, nitrotyrosine formation, NF-κB activation, pro-inflammatory cytokine concentrations, and attenuated histological injury in murine acute lung injury. We can only speculate on this discrepancy, but these authors used a short-term, un-resuscitated, sterile model in spontaneously breathing mice, whereas we investigated resuscitated polymicrobial septic shock in anesthetized and mechanically ventilated animals. At first glance, the reduced cytokine concentrations seem to be in contrast with the unchanged NF-κB activation. The timing of the measurements most likely matters in this context: We obtained tissue samples at 20 to 22 h after induction of CLP, and we previously demonstrated that NF-κB activation was increased at 3 and 6 h and subsequently decreased at 12 and 36 h after CLP induction [33]. It is well established that NF-κB activation triggers not only inflammation but also its resolution [34], and the present finding well agrees with previous data on the effect of combining Na_2_S and hypothermia in the same model: the lowest pro-inflammatory cytokine plasma and tissue concentrations and the highest blood IL-10 levels coincided with the maximum NF-κB activation [27].

Interestingly, HAM1101 reduced iNOS expression and nitrotyrosine formation but did not affect vascular eNOS expression. Moreover, ADM plasma concentrations were not significantly reduced either, the mean value in the HAM1101-treated animals being five times higher than in mice without surgery. This finding fits with Gupta et al. reporting that the salutary effect of activated protein C in rats was not associated with complete reversal of the LPS-induced increase in blood NO and ADM levels [15]. It is tempting to speculate that HAM1101 attenuated excess NO and ADM formation rather than completely blocking it while maintaining the constitutive production necessary for organ homoeostasis [9, 12]. Clearly, the attenuated iNOS activation is not mirrored by reduced NO_2_^−^ + NO_3_^−^ levels. Because of the limited blood volume available, these values were only obtained at the end of the experiment, i.e., at approximately 22 h after CLP induction. Hence, this finding well fits with previous studies by others and ourselves that NO_2_^−^ + NO_3_^−^ concentrations were comparable at 18 to 24 h post-CLP in mice with ascorbate- [35] or GW274150-induced iNOS inhibition [29] and peroxynitrite blockade [36].

We did not measure the degree of interstitial edema; however, the threefold higher urine output together with the identical fluid administration rates and total hemoglobin content suggest that HAM1101 attenuated a possible ADM-related fluid extravasation [37]. Consequently, HAM1101 most likely allowed for better maintenance of circulating blood volume. The higher urine output coincided with improved creatinine clearance and lower blood creatinine, urea, and NGAL levels. This evidence of improved glomerular and tubular function [38] was associated with reduced iNOS expression and nitrotyrosine formation, a well-established marker of peroxynitrite formation. Both iNOS activation and peroxynitrite production play a crucial role for acute kidney injury: selective iNOS blockade and catalytic peroxynitrite decomposition attenuated kidney dysfunction and organ damage in murine CLP-induced sepsis [36, 39, 40]. It could be argued that the improved renal function is not mirrored by kidney histology: in fact, tubular necrosis was absent, and glomerular tubularization was only moderate. Nonetheless, the reduced expression of activated caspase 3 suggests less apoptosis. Moreover, we investigated anesthetized and mechanically ventilated mice receiving fluid resuscitation and noradrenaline to maintain normotensive hemodynamics: in un-resuscitated murine CLP-induced septic shock of comparable duration (18 to 20 h), histological damage coincided with five- to tenfold higher creatinine and urea blood levels [41]. Finally, more advanced glomerular tubularization was present only after 2 to 3 days and severe kidney dysfunction with at least ten times higher creatinine levels [38].

Albeit noradrenaline infusion rates were lower; HAM1101 was associated with comparable endogenous glucose production. Together with the reduced iNOS expression, this finding is in line with our previous study in mice with genetic deletion and selective pharmacological inhibition of iNOS: hepatic gluconeogenesis was comparable despite lower noradrenaline infusion rates, indicating improved hepatic metabolic performance [42]. HAM1101 was associated not only with well-maintained glucose formation but also with a higher direct, aerobic glucose oxidation rate. We could not measure whole-body O_2_ uptake, but together with the identical total CO_2_ production and the comparable endogenous glucose production rate, this finding indicates that HAM1101 caused a preferential use of glucose for energy metabolism. Such a switch in fuel utilization is associated with an improved yield of oxidative phosphorylation [43]: The ratio of ATP synthesis/oxygen consumption ratio is higher for glycolysis than for β-oxidation because nicotinamide adenine dinucleotide (NADH) as an electron donor provides three coupling sites rather than two only from flavin adenine dinucleotide (FADH_2_) [44].

### Limitations of the model

HAM1101 was administered immediately after the CLP procedure. Hence, HAM1101 may only be efficacient when administered during the very early phase of sepsis. Consequently, further studies are warranted on the time dependency of the HAM1101 treatment.

All mice needed noradrenaline to maintain target hemodynamics. Consequently, any beneficial effects of HAM1101 may have been caused by ADM binding per se or due to this reduction in catecholamine requirements: noradrenaline may exert cardio- and hepatotoxic effects, enhance systemic inflammation and promote oxidative stress, and impair mitochondrial respiration.

## Conclusions

During resuscitated, polymicrobial, murine septic shock, early ADM binding using the newly developed N-terminal anti-ADM antibody HAM1101 improved catecholamine responsiveness and blunted the shock-related impairment of energy metabolism ultimately coinciding with attenuated kidney dysfunction and organ injury. These beneficial effects of HAM1101 resulted from attenuation of the systemic inflammatory response and reduced nitrosative stress while constitutive NO synthesis was unaffected.

## Electronic supplementary material

Additional file 1: **Supplement.** Adrenomedullin (ADM) and ADM antibody (HAM1101) preparation. (DOC 230 KB)
