# Supplementary material for: Adrenomedullin binding improves catecholamine responsiveness and kidney function in resuscitated murine septic shock
Source: Intensive Care Med Exp. 2013 Oct 29;1:2. doi: 10.1186/2197-425X-1-2 (PMC4796991; doi:10.1186/2197-425X-1-2)
Supplement: Supplementary file 1 — Additional file 1: Supplement. Adrenomedullin (ADM) and ADM antibody (HAM1101) preparation. (DOC 230 KB) [file 40635_2013_21_MOESM1_ESM.doc]

***Supplement:***

**Adrenomedullin (ADM) and ADM antibody (HAM1101) preparation**

*Generation of Antibodies:*

Mouse Adrenomedullin (ADM) peptides for immunization were synthesized (JPT Technologies, Berlin, Germany) with an additional N-terminal Cysteine (if no cysteine is present within the selected ADM-sequence) residue for conjugation of the peptides to bovine serum albumin (BSA). The peptides (table 1) were covalently linked to BSA by using SulfoLink-coupling gel (Perbio Science, Bonn, Germany). The coupling procedure was performed according to the manufacturer’s protocol. The monoclonal murine antibodies were generated according to standard procedures.

Table 1. ADM-derived peptides for immunization and resulting antibodies. mADM middle-adrenomedullin.

| Antigen | mADM region | Antibody name |
| --- | --- | --- |
| YRQSMNNGSRSNGC | 1-14 | HAM1101 |
| CTFQKLAHQIYQ | 19-31 | MAM1201 |
| CAPRNKISPQGY-NH_2_ | C-40-50 | MAM1301 |
| CTVQKLAHQIYQ | 21-32 | HAM2203 |

*Mouse monoclonal antibody production:*

Antibodies were produced using standard antibody production methods [Marx 1997] and purified via Protein A. The antibody purities were > 95% based on SDS gel electrophoresis analysis.

*Immunoassay for the quantification of mouse adrenomedullin:*

The technology used was a sandwich coated tube luminescence immunoassay, based on acridinium NHS-ester labelling.

*Labelled compound (tracer)*: 200 µg (333 µL) MAM1301 (0.6 mg·mL^-1^ PBS pH 7.4) was mixed with 10 µL acridinium NHS-ester (1 mg·mL^-1^ in acetonitrile, InVent, Braunschweig, Germany) and incubated for 20 minutes at 21-25 °C. After adding 50 µL glycine (50mM) MAM1301 was purified by Gel-filtration HPLC on Bio-Sil® SEC 400-5 (Bio-Rad Laboratories Inc., Hercules, CA). The purified MAM1301 was diluted in (300 mmol·L^-1^ potassiumphosphate, 100 mmol·L^-1^ NaCl, 10 mmol·L^-1^ Na-EDTA, 5 g·L^-1^ BSA pH 7.0). The final concentration was approx. 800.000 relative light units (RLU) of labelled compound (approx. 20 ng labelled antibody) per 200 µL acridinium NHS-ester. Chemiluminescence was measured by using an AutoLumat LB 953 (Berthold Technologies, Bad Wildbad, Germany).

*Solid phase:* Polystyrene tubes (Greiner Bio-One, Kremsmünster, Austria) were coated (18 hours at 21-25 °C) with MAM1201 (1.5 µg in 0.3 mL, 50 mmol·L^-1^ TRIS/HCl, 100 mmol·L^-1^ NaCl, pH 7.8). After blocking with 5% BSA, the tubes were washed with PBS pH 7.4, and vacuum dried.

*Calibration:* The assay was calibrated, using dilutions of mADM (Bachem, Bubendorf, Switzerland) in Sample Diluent (250 mmol·L^-1^ NaCl, 2 g·L^-1^ g/L Triton X-100, 50 g·L^-1^ BSA, 20 tabs per L Protease Inhibitor Cocktail pH 7.8; Roche Diagnostics, Basel, Switzerland).

*mADM Immunoassay:* 50 µL of sample (or calibrator) was pipeted into coated tubes. After adding labeled MAM1301 (200 µL), the tubes were incubated 20 h at 2-8°C. Unbound tracer was removed by washing 5 times (1 mL each) with wash solution (20mM PBS, pH 7.4, 0.1 % Triton X-100). Tube-bound chemiluminescence was measured using the LB 953 Luminometer.

Figure 1. Typical ADM dose/signal curve and an ADM dose/signal curve in the presence of 10 µg·mL^-1^ (final assay volume) antibody HAM1101. The one-step assay format (mean values of duplicate determination, CV <10%) uses MAM1201 as tube and MAM1301 as tracer antibody. 50 µL of standard material and 200 µl tracer were incubated 20 h at 2-8 ° C, washed and measured by using the LB 953 Luminometer. HAM1101 did not interfere with the described mADM immunoassay.

*Immunoassay for the quantification of HAM1101:*

The technology used was a sandwich coated tube luminescence immunoassay, based on acridinium NHS-ester labelling.

*Labelled compound (tracer):* HAM1101 was labeled according to the above described procedure.

*Solid phase:* HAM2203 coated polystyrene tubes were prepared as described above.

*Calibration:* The assay was calibrated, using dilutions of HAM1101 in Sample Diluent.

*HAM1101 Immunoassay:* Samples were diluted 1:25 in Sample Diluent. 50 µL of sample (or calibrator) were pipetted into coated tubes. After adding 100 µL of 10 ng·mL^-1^ hADM (BACHEM) sample diluent, the tubes were incubated for 1 hour at 2-8 °C. After the incubation labelled HAM1101 (100 µL) was added, and subsequently incubated for 3 hours at 2-8° C. Unbound tracer was removed by washing 5 times (1 mL each) with wash solution. Tube-bound chemiluminescence was measured by using a LB 953 Luminometer.

Figure 2. Typical HAM1101 dose/signal curve. The two-step assay format (mean values of duplicate determination, CV < 10%) uses the antibodies HAM2203 as tube and HAM1101 as tracer antibody. 50 µL of standard material and 100 µl human ADM (10 ng·mL^-1^ final assay volume) were incubated for 1 hour at 2-8 °C. Thereafter, 100 µL tracer were added and incubated for 3 hours at 2-8 °C, washed and measured using the LB 953 Luminometer.

*Characterization of HAM1101:*

Affinity was determined by using the Biacore Technology (Biaffin, Kassel, Germany). The affinity constant of HAM1101 towards human ADM was 3.9·10^-9^ M^-1^.

*Immunoassay for the quantification of HAM1101 specificity:*

The technology used was a peptide coated tube luminescence immunoassay, based on acridinium NHS-ester labelling.

*Labelled compound (tracer):* HAM1101 was labeled according to the above described procedure.

*Solid phase:* Polystyrene tubes (Greiner Bio-One) were coated (18 hours at 21-25 °C) with Streptavidin from *Streptomeyces avidinii* (Sigma Aldrich, St. Louis, MO; 2 µg in 0.3 mL), 50 mmol·L^-1^ TRIS/HCl, 100 mmol·L^-1^ NaCl pH 7.8). After blocking with 5% BSAe, the tubes were washed with PBS pH 7.4. Streptavidin-coated tubes were incubated for 1 hour at 21-25 °C with ADM_1-14_-Biotin (JPT; 10 ng in 0.3 mL), 50 mmol·L^-1^ TRIS/HCl, 100 mmol·L^-1^ NaCl pH 7.8). After 2 times washing with PBS pH 7.4, the tubes were blocked with 5% BSAe and vacuum dried.

*HAM1101 specificity Immunoassay:* First 100 µL of sample were pre-incubated with labeled HAM1101 (200 µL) for 1 hour at 21-25 °C. Afterwards 250 µL of the mixture were pipetted into solid phase, and incubated for 20 hours at 2-8 °C. Unbound tracer was removed by washing 5 times (1 mL each) with wash solution. Tube-bound chemiluminescence was measured by using a LB 953 Luminometer.

**References:**

Marx U, Embleton J, Fischer R, et al: Monoclonal antibody production. The report and recommendations of ECVAM workshop. *ATLA* 1997;25:121-137
